# The prevalence of and major risk factors associated with diabetic retinopathy in Gegharkunik province of Armenia: cross-sectional study

**DOI:** 10.1186/s12886-015-0032-0

**Published:** 2015-04-30

**Authors:** Aida Giloyan, Tsovinar Harutyunyan, Varduhi Petrosyan

**Affiliations:** Garo Meghrigian Institute for Preventive Ophthalmology, School of Public Health, American University of Armenia, 40 Marshal Baghramian Ave., Yerevan, 0019 Armenia; School of Public Health, American University of Armenia, 40 Marshal Baghramian Ave., Yerevan, 0019 Armenia

**Keywords:** Diabetes, Diabetic retinopathy, Risk factors, Diabetes duration and Insulin treatment

## Abstract

**Background:**

Diabetic retinopathy (DR) is one of the leading causes of blindness in adults in industrialized countries and the emerging cause of blindness in developing countries. The objective of this study was to describe the prevalence of DR and risk factors associated with it among diabetic patients.

**Methods:**

The analytical cross-sectional survey and eye screenings were carried out among 625 diabetic patients from urban and rural areas of Gegharkunik region. DR was assessed by dilated ophthalmoscopy and defined based on the WHO International Classification of Diseases. The survey instrument, included questions about demographics, disease history, health status, medication use and healthy lifestyle. Descriptive statistics and logistic regression were used to analyze the data.

**Results:**

The prevalence of DR in the sample was 36.2%. A total of 90.2% of patients with DR had non-proliferative, while 9.8% had proliferative DR. In bivariate analysis, age, diabetes duration, being under insulin treatment, blood glucose level, having non-communicable diseases were significantly associated with DR. In the adjusted analysis being under insulin treatment (OR = 3.24; 95% CI: 1.56–6.75), diabetes duration (OR = 1.23; 95% CI: 1.16–1.31) and age (OR = 1.05; 95% CI: 1.02–1.08) were independently associated with DR.

**Conclusion:**

Earlier diagnosis of diabetes and DR can help to control some of these factors and prevent further complications and vision loss. Population-based educational programs on diabetes and diabetic retinopathy and continuous medical education on diabetes management can improve diabetes care and self-management and prevent eye complications.

## Background

Visual impairment and blindness are major public health problems causing significant suffering, disability, loss of productivity, and diminishing quality of life for millions of people. Vision disability is one of the top 10 disabilities among adults 18 years and older worldwide [1]. Age-related blindness is increasing throughout the world, as is blindness due to uncontrolled diabetes [2]. Uncontrolled hyperglycaemia in diabetic patients can lead to significant and widespread pathological changes, including damage to the retina, brain and kidney [3]. After 15 years of diabetes, approximately 2% of people become blind, and about 10% develop severe visual impairment [4]. After 20 years, more than 75% of patients will have some form of diabetic retinopathy [5].

Diabetic retinopathy (DR) is a well-recognized complication of diabetes mellitus that occurs as a result of long-term accumulated damage to the small blood vessels in the retina [4,5].

Diabetic retinopathy (DR) is one of the leading causes of blindness in adults of working age (20 to 65 years) in industrialized countries and the emerging cause of blindness in developing countries [3,4]. More than 2.5 million people worldwide are affected by DR. Clinical trials have shown that good control of diabetes and hypertension significantly reduces the risk for diabetic retinopathy [5]. If DR has already developed, timely treatment of retinopathy can reduce the risk for visual loss by more than 90%; however, the awareness among patients with diabetes about diabetic retinopathy is limited, and compliance with treatment is often poor [5].

The strongest risk factors for DR that are consistently described in the literature include diabetes duration [6-12], glycemic control [13-15], insulin treatment [6,11,12,15] and hypertension [7-9,16-18]. Associations of DR with type of diabetes [3,9,19], age [9,12,16,17], age at diabetes onset [6,9], high levels of serum cholesterol and /or triglycerides [12,16], obesity [8,13], smoking [7,9], physical activity [20,21] and gender [7,17] have also been documented [3,6-9,12,13,16,17,19]. Since some of the risk factors are modifiable, regular screenings for these factors in people with diabetes might help to develop timely management strategies and reduce complications and vision loss.

Diabetes-related morbidity, disability and mortality are growing public health concerns in Armenia. According to the World Health Organization (WHO) the diabetes mortality rate in Armenia was 46.5 deaths per 100 000 population in 2009 [22]. According to the International Diabetes Federation (IDF) estimates, the diabetes morbidity rate is projected to increase by 0.9% annually in Armenia from 2010 to 2030; which means every tenth citizen in Armenia would have diabetes in 2030 [22]. Studies of prevalence and risk factors for different eye pathologies in Armenia are scarce. The only study that investigated the prevalence and risk factors associated with different eye conditions in Armenia was conducted in 2004 by Garo Meghrigian Institute for Preventive Ophthalmology (GMIPO) [23]. The population based study of people 50 years old and over showed that 3.7% were blind with 0.5% having bilateral blindness, and 3.2% unilateral [23]. The main causes of blindness and visual impairment were cataract, glaucoma, diabetic retinopathy (9.1% of all bilateral blind cases) and uncorrected refractive errors [23]. The overall prevalence of diabetic retinopathy was 0.7% among the screened population.

The main obstacles for effective diabetes management in the country were the limited supply of anti-diabetic drugs, which placed a heavy financial burden on diabetes patients; changes in the types and brands of the anti-diabetic drugs, which lead to destabilization of patients’ blood glucose level; high price of hospital care and advanced laboratory testing; lack of access to consumables (glucometers, strips and syringes) and patients’ lack of knowledge which significantly reduce the effectiveness of diabetes self-management [22].

Other underlying factors associated with poor management of diabetes patients was lack of diabetes screening programs, shortages of endocrinologists in more remote regions of Armenia and high social stigma associated with diabetes and insulin-use, especially among younger diabetes patients. Absence of comprehensive diabetes registry and lack of coordination of care between primary, secondary and tertiary level medical centers threatens the quality of diabetes care and puts patients at greater risk for complications [22].

The aim of the current study was to describe the prevalence of diabetic retinopathy and risk factors associated with it in diabetic patients in Gegharkunik province, Armenia.

## Methods

### Data collection

Diabetic patients from urban and rural areas in Armenia register in primary health care (PHC) facilities for regular care and for receiving state guaranteed diabetes treatment [22]. All registered diabetic patients from five cities (Sevan, Gavar, Martuni, Tchambarak and Vardenis) and 56 out of 87 villages in Gegharkunik province were invited to their respective facilities for ophthalmic examination with the focus on detection of diabetic retinopathy. The endocrinologists working at the primary health care facilities invited the patients for screenings by phone. From 1,329 registered diabetics in Gegharkunik province 625 participated in the eye screenings and interviews. The response rate was 47.0%. Data were collected during July - December, 2012.

### Instrument

The research team developed a structured questionnaire for face to face interviews with diabetic patients. The questionnaire included questions about demographic data, disease history, health status in terms of having chronic conditions, family history of diabetes, and health-related behaviors. The research team used the Diabetes Specific Scale designed by Stanford Patients Education Research Center [24] as a base for developing the questionnaire. The questionnaire was adapted, translated and pre-tested for use in Armenian population.

### Study variables

The main outcome variable of interest in this study was the presence of *diabetic retinopathy* (dichotomous). Independent variables included age (continuous), gender (dichotomous), urban/rural residence (dichotomous), education (categorized as “incomplete secondary”, “secondary”, “incomplete college/university” and “university/college”), type of diabetes (dichotomous), participants’ body mass index (BMI kg/m [2]) (categorized as normal weight (BMI: 18.5-24.9), overweight (BMI: 25.0-29.9), mild or class I obesity (BMI: 30.0-34.9), moderate or class II obesity (BMI: 35.0-39.9) and severe or class III obesity (BMI: >40)) [25], duration of diabetes in years (continuous), being under insulin treatment (dichotomous), presence of chronic non-communicable diseases such as hypertension, high cholesterol level, heart diseases, and renal diseases (dichotomous), blood glucose level (continuous), use of medication for controlling diabetes and blood pressure (dichotomous), current smoking status (dichotomous), and physical activity (dichotomous).

The *blood glucose level* was estimated based on the patients’ recall of the latest result of their blood glucose level.

*Use of diabetes medication* was assessed by asking whether the patient took pills for diabetes in the past week and *use of blood pressure medication* was assessed by asking whether the patient took pills to control high blood pressure in the past week.

*Physical activity* was assessed based on the physical activity scale from the Behavioral Risk Factors Surveillance System Questionnaire [26]. Participants were classified as physically active if they were moderately active for at least five days per week with at least 30 minutes per day, based on recommendations from the Centers for Disease Control and Prevention (CDC) and the American College of Sports Medicine [27,28].

### Eye screening procedure

All participants underwent detailed ophthalmologic screening examination, including measurements of visual acuity by Golovin-Sivtsev chart, cycloplegic skiascopy, measurements of intraocular eye pressure (IOP) and dilated eye fundus examination. Eye screenings were carries out by the GMIPO team, which consists of two experienced ophthalmologists, a nurse and an interviewer. DR was assessed by dilated ophthalmoscopy and defined based on the WHO International Classification of Diseases [29]. Nonproliferative diabetic retinopathy was diagnosed if microaneurysm, hemorrhages, soft exudates, venous beading and intraretinal microvascular abnormalities were identified in retina. Pre-proliferative diabetic retinopathy was diagnosed if new vessels were identified in retina and definition not met for proliferative retinopathy. Proliferative diabetic retinopathy was diagnosed based on the formation of new vessels at the disk (NVD) and elsewhere (NVE). New vessels occur on or within optic disk about one-quarter to one-third disk area, with or without vitreous or pre-retinal hemorrhage. New vessels occur along the border between healthy retina and areas of capillary occlusion, on the iris and on the anterior hyaloid surface [30,31]. Diabetic angiopathy was diagnosed if retinal ischemia caused by capillary occlusion, retinal neovascularization and microaneurysms were identified in retina [32].

### Ethical consideration

The study received approval from Institutional Review Board of the American University of Armenia. Oral consent was obtained from the patients. This study adhered to the guidelines of the Declaration of Helsinki.

### Analysis

The study team analyzed associations between risk factors and having DR using logistic regression analysis. Bivariate logistic regression identified factors significantly associated with DR. The multivariate analysis included all variables that were found to be associated with DR at the p < 0.25 level in the bivariate analysis [33] and the factors which have been shown to be important for the development of DR in the literature.

Analysis included descriptive statistics, unadjusted logistic regression, multivariate logistic regression and testing of collinearity. Data were analyzed using SPSS version 17.0 (SPSS Inc., Chicago, IL, USA).

## Results

### Demographic characteristics of respondents

Mean age of participants was 61.34 (SD = 11.01), ranging from 15 to 88 years. Females comprised 64.3% of the sample. We compared the age and sex distribution of our sample to that of the total population of diabetics in Gegharkunik province. The mean age of all diabetic patients in Gegharkunik province was 59.99 (SD = 11.17) and females comprised 57.6% of that population.

About 11.4% of the sample had higher education (university/college), 17.5% had “incomplete university/college”, 47.7% had “secondary”, and 23.3% had “incomplete secondary” education. Overall, 43.2% of participants were from urban and 56.8% from rural areas.

### Prevalence of DR and other health status characteristics

The overall prevalence of DR in the sample was 36.2% (Table 1). Age and gender standardized prevalence of DR considering all registered patients with diabetes in Gegharkunik province was 35.4%. A total of 90.2% of the patients with DR had non-proliferative DR, and 9.8% of them had proliferative DR. Pre-proliferative DR (moderately to very severe non-proliferative DR) was diagnosed in 31.7% of 202 individuals with non-proliferative DR. Diabetic angiopathy was prevalent in 18.4% of diabetic patients. Eleven percent of diabetic patients had macular degeneration.

**Table 1:** **Respondents’ health status characteristics**

	**% (N)**	**95% CI**
**Diabetic Retinopathy**	36.2 (224)	(32; 40)
Non- proliferative	90.2 (202)	(86; 94)
*Pre-proliferative*	*31.7 (64)*	*(25; 38)*
Proliferative	9.8 (22)	(96; 99)
**Eye diseases**		
Macular degeneration	11.0 (69)	(8;13)
Diabetic angiopathy	18.4 (115)	(15; 21)
**Types of diabetes**		
Type 1	2.9 (18)	(2; 4)
Type 2	34.2 (214)	(30; 38)
Do not know	62.9 (393)	(59; 67)
**Years of having diabetes**	Mean (SD)	
	7 (6.0)	
**BMI** *(kg/m* ^*2*^ *)*		
Normal weight	17.9 (104)	(15; 21)
Overweight	35.3 (205)	(31; 39)
Mild or class I obesity	32.9 (191)	(0.29; 0.37)
Moderate or class II obesity	10.8 (63)	(0.08; 0.13)
Severe or class III obesity	3.1 (18)	(0.02; 0.04)
**Family history of having diabetes**		
Yes	53.1 (330)	(49; 57)
No	46.9 (292)	(43; 50)
**Chronic disease**		
Hypertension	66.3 (413)	(63; 70)
Heart disease	43.3 (270)	(40; 47)
Renal disease	28.7 (179)	(25; 32)
High cholesterol level	22.0 (137)	(20; 25)
**Self-reported health**		
Excellent	0.6 (4)	(0.3; 0.9)
Very good	0.3 (2)	(−0.1; 0.7)
Good	14.3 (89)	(11; 17)
Satisfactory	58.4 (363)	(55; 61)
Bad	26.4 (164)	(23; 30)

About 62.9% of the patients did not know about the type of diabetes they had. Among those who knew, 2.9% had type 1, and 34.2% had type 2 diabetes. On average, patients have had diabetes for 7 years (SD = 6.0). About 17.9% of the respondents had normal weight, 35.3% were overweight, 32.9% had mild or class I obesity, 10.8% had moderate or class II obesity and 3.1% had severe or class III obesity. About 53% of respondents mentioned having diabetes among first degree relatives.

Hypertension was the most prevalent chronic condition, with 66.3% of the respondents suffering from it. High cholesterol level was reported by 22.0%. Most of the patients (58.4%) described their overall health status as “satisfactory”, 26.4% described it as “bad”, 14.3% as “good”, 0.6% as “excellent”, and 0.3% as “very good”.

### Self-reported use of medication, physical activity and smoking

Ninety six percent of the study population reported that they took pills to control diabetes in the past week and 84.2% of study participants reported taking pills to control high blood pressure in the past week. About half of the patients (51.2%) were physically active and only 8.0% reported smoking currently.

### Logistic regression analysis of risk factors associated with diabetic retinopathy

The bivariate logistic regression showed that age, being overweight, diabetes duration, being under insulin treatment, blood glucose level, having non-communicable diseases (hypertension, renal and heart diseases) were significantly associated with DR. Unadjusted analysis showed no significant association between DR and type of diabetes, gender, age at the onset of diabetes, BMI, high cholesterol level, use of medication for controlling diabetes, smoking, and physical activity.

In the adjusted analysis age, diabetes duration and being under insulin treatment were associated with diabetic retinopathy (Table 2). The odds of having DR increased by 5.0% with each additional year of age (OR = 1.05; 95% CI: 1.02 – 1.08) after adjusting for other covariates. Each additional year of diabetes duration was associated with 23.0% higher odds of having DR (OR = 1.23; 95% CI: 1.16 – 1.31). Adjusted odds of having DR were 3.24 times higher among those who were treated with insulin compared to patients treated with other glucose controlling medication (OR = 3.24; 95% CI: 1.56 – 6.75). The effects of other factors such as current blood glucose level, having non-communicable diseases dissipated in the adjusted model.

**Table 2:** **The risk factors for diabetic retinopathy: Results of unadjusted and adjusted logistic regression**

	**Unadjusted OR (95% CI)**	**Unadjusted p-value**	**Adjusted OR (95% CI)**	**Adjusted p-value**
Age (*in years*)	1.04 (1.02; 1.05)	0.00	1.05 (1.02; 1.08)	0.00
Gender				
Female	1.00		1.00	
Male	0.95 (0.67; 1.34)	0.76	0.96 (0.53; 1.74)	0.89
BMI (*kg/m* ^*2*^)				
Normal	1.00		1.00	
Overweight/Obese	1.23 (0.78; 1.93)	0.38	1.76 (0.86; 3.58)	0.12
Diabetes duration (*in years*)	1.23 (1.18; 1.28)	0.00	1.23 (1.16; 1.31)	0.00
Being under insulin treatment	3.36 (2.25; 5.04)	0.00	3.24 (1.56; 6.75)	0.00
Current blood glucose level	1.05 (1.02; 1.09)	0.00	0.98 (0.93; 1.05)	0.63
Non-communicable diseases*	1.83 (1.20; 2.77)	0.00	1.10 (0.57; 2.14)	0.77
Smoking	0.84 (0.45; 1.57)	0.41	0.64 (0.18; 2.22)	0.48
Physical activity	0.87 (0.57; 1.32)	0.51	1.10 (0.65; 1.88)	0.71

*Hypertension, heart diseases and renal diseases.

## Discussion

This study provides unique data on the prevalence of diabetic retinopathy and the associated factors in one of the regions of Armenia – a country for which no information on DR have been previously available in the literature. The study showed that the prevalence of diabetic retinopathy was 36.2% among diabetic patients in Gegharkunik region. The prevalence of DR in this population was similar to or lower than the rates that were found in other countries in the region, including Iran (37.0% of diabetic patients) [34] and Russia (46.0% of diabetic patients) [35]. Various studies report different rates of DR worldwide, with the estimates largely depending on the methodology, setting, diagnostic method, and the population sample [18]. The prevalence of DR among diabetic patients reported in a large study conducted in the US was 33.2% [15], while the studies conducted in Norway and the UK report substantially lower prevalence of DR, 13.0% and 29.4%, respectevily [10,11]. The study by Thomas et al. conducted in the UK among 57,199 diabetic patients reported 29.4% prevalence of DR. [11] A large meta-analysis by Joanne et al. based on data from 35 studies in different countries (1980–2008) from 22,896 individuals with diabetes showed that the global prevalence of DR was 34.6% [36], while the study by Ruta et al. showed 27.9% (22-37%) median prevalence of diabetic retinopathy in 33 developed and developing countries [37]. The slightly higher prevalence of DR in Armenia might be explained by lack of preventive efforts among Armenian diabetic patients such as regular screenings and patient counseling about diabetic retinopathy and its prevention and control. These problems might be typical for other countries of Eastern Europe, where lack of systematic screenings for eye complications in diabetes patients, particularly in rural areas, has been highlighted [38,39].

The risk factors that were shown to have significant and independent associations with diabetic retinopathy in the multivariate analysis in this study included age, duration of diabetes and being under insulin treatment.

The duration of diabetes in the present study was strongly associated with DR. About 76.7% of patients who suffered from diabetes for more than 20 years were diagnosed with DR. Aiello et al. reported that after 20 years of diabetes, more than 60 percent of type 2 diabetes patients will have retinopathy regardless of the diabetic control [40]. This have been confirmed by numerous other studies [9,10,12,14]. Studies suggest that duration of diabetes might reflect total glycemic control and exposure to other risk factors over time [18,40].

With age, chances of getting DR increase in diabetic patients; [17] also, the increasing age contributes significantly to the severity of DR [6,12]. In this study we recorded limited effect of age with marginal significance. Some authors suggest that age might be a surrogate marker of duration of DR in patients [41].

Being under insulin treatment was the strongest risk factor for DR among the participants of this study, with odds of having DR being 2.35 times higher among those who were treated with insulin compared to patients treated with other glucose controlling medication. This finding was consistent with other studies [6,11,12,42]. Although some authors assume that being on insulin treatment might be related to the duration of the disease [43], in our study the effect of insulin was present even after controlling for the duration of diabetes. Younis et al. reported that patients using insulin were at higher risk of progression to sight-threatening diabetic retinopathy in the longitudinal Liverpool Diabetic Eye Study [42]. The authors suggested that the need for insulin treatment might reflect poorer metabolic control in such patients.

Several epidemiological studies examined the relationship of BMI and DR and found inconsistent results [8,13,41,44-48]. Most studies show positive association between high BMI or obesity with DR [8,13,44], while other studies reported contradictory findings [41,45-48]. Our study found positive but not statistically significant association between overweight/obesity with DR.

Having chronic non-communicable diseases (hypertension, renal and heart diseases) and self-reported high blood glucose level were not significantly associated with DR in the multivariate analysis. Most studies show that high blood pressure is significantly associated with DR [7,9,17]. It is possible that our analysis failed to reveal the association between chronic non-communicable diseases and DR because we used self-reported data on these conditions instead of the data obtained from medical records. The same concern is valid for the blood glucose level. In our study, the patients were asked to recall the latest result of their blood glucose level; however, their recall might have been inaccurate and might have biased the results of the analysis. Our decision to rely on self-reported data was largely conditioned by the difficulties to obtain reliable and detailed records on the indicators of interest from the local primary healthcare facilities.

Our study has several limitations. First, the prevalence statistics reported in our study is based on the sample of diabetic patients who were registered in primary healthcare facilities in Gegharkunik province and who agreed to participate in the screening with the response rate of 47%. It is possible that those who refused or were unable to participate were systematically different from those who were included in the study, particularly in terms of their diabetes status and vision problems. Second, our study used clinical examination for diagnosing cases with diabetic retinopathy instead of performing stereoscopic fundus photography and did not assess inter- and intra-observer agreement on diagnoses of diabetic retinopathy.

Some of the risk factor variables which were used in the study, including health status, chronic non-communicable diseases, blood glucose level, years of having diabetes, smoking and physical activities were based on self-report of the patients due to absence of quality measurements from local laboratories for all patients who were involved in the study, which might have introduced a recall bias.

## Conclusion

This study found high prevalence of DR among Armenian diabetic patients. Age, diabetes duration, being overweight and being under insulin treatment were independently associated with diabetic retinopathy. Our findings warrant increased efforts by policy makers and health care providers in Armenia to ensure timely diagnosis of DR and better control of factors associated with DR among diabetic patients.

Population-based educational programs on diabetes and diabetic retinopathy and continuous medical education trainings in diabetes management can improve diabetes care and self-management and prevent eye complications.

## Abbreviations

BMI: Body mass index
CDC: Centers for disease control and prevention
DR: Diabetic retinopathy
IDF: International diabetes federation
IOP: Intraocular eye pressure
NVD: New vessels at the disk
NVE: New vessels elsewhere
WHO: World health organization
